# Novel Insights Into the Association Between Parkinson's Disease and Constipation: Role of SHMT2 as a Promising Biomarker

**DOI:** 10.1002/cns.70912

**Published:** 2026-05-03

**Authors:** Jiehua Su, Kaixun Huang, Xiuna Jing, Xiaohuan Liu, Cheng Wen, Rulin Geng, Zhuoying Lai, Zhijia Ruan, Yiqiang Zhan, Danyu Lin, Enxiang Tao

**Affiliations:** ^1^ The Department of Neurology The Eighth Affiliated Hospital, Sun Yat‐Sen University Shenzhen China; ^2^ The Department of Neurology Sun Yat‐Sen Memorial Hospital, Sun Yat‐Sen University Guangzhou China; ^3^ Department of Epidemiology, School of Public Health (Shenzhen) Sun Yat‐Sen University Shenzhen China

**Keywords:** bioinformatics analysis, biomarker, constipation, Parkinson's disease, SHMT2

## Abstract

**Aims:**

We aimed to investigate the shared molecular pathways between Parkinson's disease (PD) and constipation using bioinformatics analysis.

**Methods:**

Differentially expressed genes (DEGs) were identified via the R limma package, and Weighted Gene Co‐Expression Network Analysis (WGCNA) was conducted to identify hub modules. Biological enrichment analysis clarified the biological processes involved. A gene–gene interaction (GGI) network was constructed to identify shared hub biomarkers. Validation of these biomarkers was done in vitro with α‐synuclein (α‐syn)‐treated SH‐SY5Y cells and in vivo through α‐syn‐induced PD mouse models, A53T transgenic mice, and loperamide‐induced constipation models.

**Results:**

Numerous DEGs were identified in both conditions, with 14 shared DEGs found through the intersection of core modules and upregulated DEGs. These DEGs are primarily involved in energy metabolism, protein modification, and mitochondrial function. Five key hub genes were identified using the GGI network and gene topological analysis. Notably, Serine hydroxymethyltransferase 2 (SHMT2) expression was significantly upregulated after α‐syn treatment in vitro. Immunohistochemical and immunofluorescence analyses revealed elevated SHMT2 expression in brain and colon tissues in PD mouse models (*p* < 0.001), whereas in constipation models, SHMT2 was only elevated in the colon wall with no significant expression in the enteric nervous system.

**Conclusion:**

Our findings offer new views on the molecular link between PD and constipation, suggesting SHMT2 as a possible biomarker and therapeutic target for PD symptoms.

## Graphical abstract

This study integrated bioinformatics analysis, cellular models, and PD/constipation mouse models to identify SHMT2 as a key shared biomarker linking Parkinson's disease and constipation. SHMT2 upregulation was validated in dopaminergic neurons and the enteric nervous system, highlighting its potential role in gut–brain axis dysfunction. These findings suggest SHMT2 as a promising diagnostic and therapeutic target for PD‐related gastrointestinal symptoms.

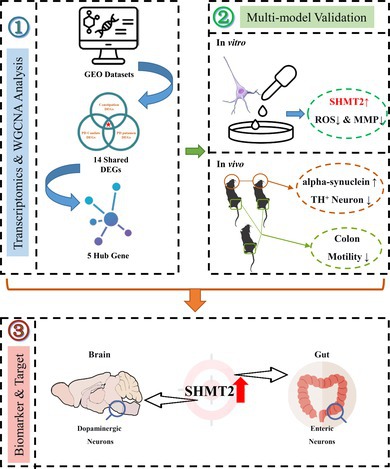

## 1 Introduction

Parkinson's disease (PD) is a multifaceted neurodegenerative disorder characterized not only by its well‐documented motor symptoms but also by significant non‐motor manifestations, including autonomic dysfunction, sleep disturbances, hyposmia, and constipation. Notably, constipation affects up to 90% of PD patients [1]. Constipation not only represents a prevalent symptom but also serves as a prodromal feature, with studies showing a 2.27‐fold increased risk of PD in individuals with chronic constipation [2]. Furthermore, constipation has been linked to a worse disease trajectory in PD, with patients experiencing major constipation at disease onset having a faster progression to dementia, postural instability, and death [3]. With its high prevalence and significant impact on disease progression, constipation is an important area of research in the field of PD, and understanding its relationship with PD pathogenesis and progression is crucial for the development of effective therapeutic strategies.

Elucidating the pathogenesis of PD is crucial for grasping the comprehensive nature of the disease, particularly in understanding how symptomatic factors like constipation offer insights into disease progression. The pathogenesis of PD is multifaceted, characterized by the aberrant aggregation of α‐synuclein (α‐syn), mitochondrial dysfunction, lysosomal impairment, and neuroinflammation—interrelated processes that likely contribute to both motor and non‐motor symptoms experienced by patients. Genetic mutations, oxidative stress, and immune activation further exacerbate these pathological processes, driving disease progression [4]. The hallmark pathological features of PD include Lewy bodies and Lewy neurites, which are primarily composed of aggregated α‐syn [5]. While the exact origins and mechanisms underlying the formation of pathogenic α‐syn remain under investigation [6], misfolded α‐syn has been identified in both the central nervous system (CNS) and the enteric nervous system (ENS) of PD patients. The dual presence of misfolded α‐syn in both systems suggests a potential link between gastrointestinal dysfunction and the pathogenesis of PD. Recent research has underscored the critical importance of the microbiome‐gut‐brain axis in PD [7], which encompasses intricate interactions among the gut microbiota, gastrointestinal function, and the CNS. Consequently, disruptions within this axis, as evidenced by constipation, have the potential to adversely impact both gastrointestinal and neurological health, thereby highlighting the interconnected nature of PD‐related symptoms. In summary, the relationship between PD and constipation may be more than coincidental, with potential overlap and interplay between their underlying pathogenic mechanisms, warranting further investigation to uncover novel insights into PD's etiology and therapeutic strategies.

Understanding the molecular mechanisms that drive constipation in PD, particularly those related to altered colonic motility or damage to the ENS [8], is essential for developing effective therapeutic strategies. However, significant gaps persist in our knowledge of these mechanisms. Current animal models, such as those utilizing α‐syn overexpression in mice [9], fall short of replicating the complexities of idiopathic PD. Notably, there is a lack of research focusing on common underlying mechanisms, molecular changes at the cellular level, and associated biomarkers bridging these two conditions. As a prototypical prodromal feature and worse disease trajectory of PD, constipation offers a unique window into the pathogenesis of the disease, and its study has the potential to reveal important insights into the underlying mechanisms of PD and the development of novel therapeutic strategies. Transcriptomic analysis offers a promising approach to identify biomarkers and shared molecular mechanisms linking constipation and PD, as it allows for the simultaneous examination of multiple genes and pathways [10]. By applying this methodology, researchers can identify key regulatory nodes and potential therapeutic targets, which may ultimately lead to the development of novel treatments for both motor and non‐motor manifestations of PD. In light of the evident and significant association between constipation and PD, we employed transcriptomic analysis to elucidate the shared molecular mechanisms and identify potential biomarkers linking these two conditions.

## 2 Materials and Methods

**FIGURE 1:**
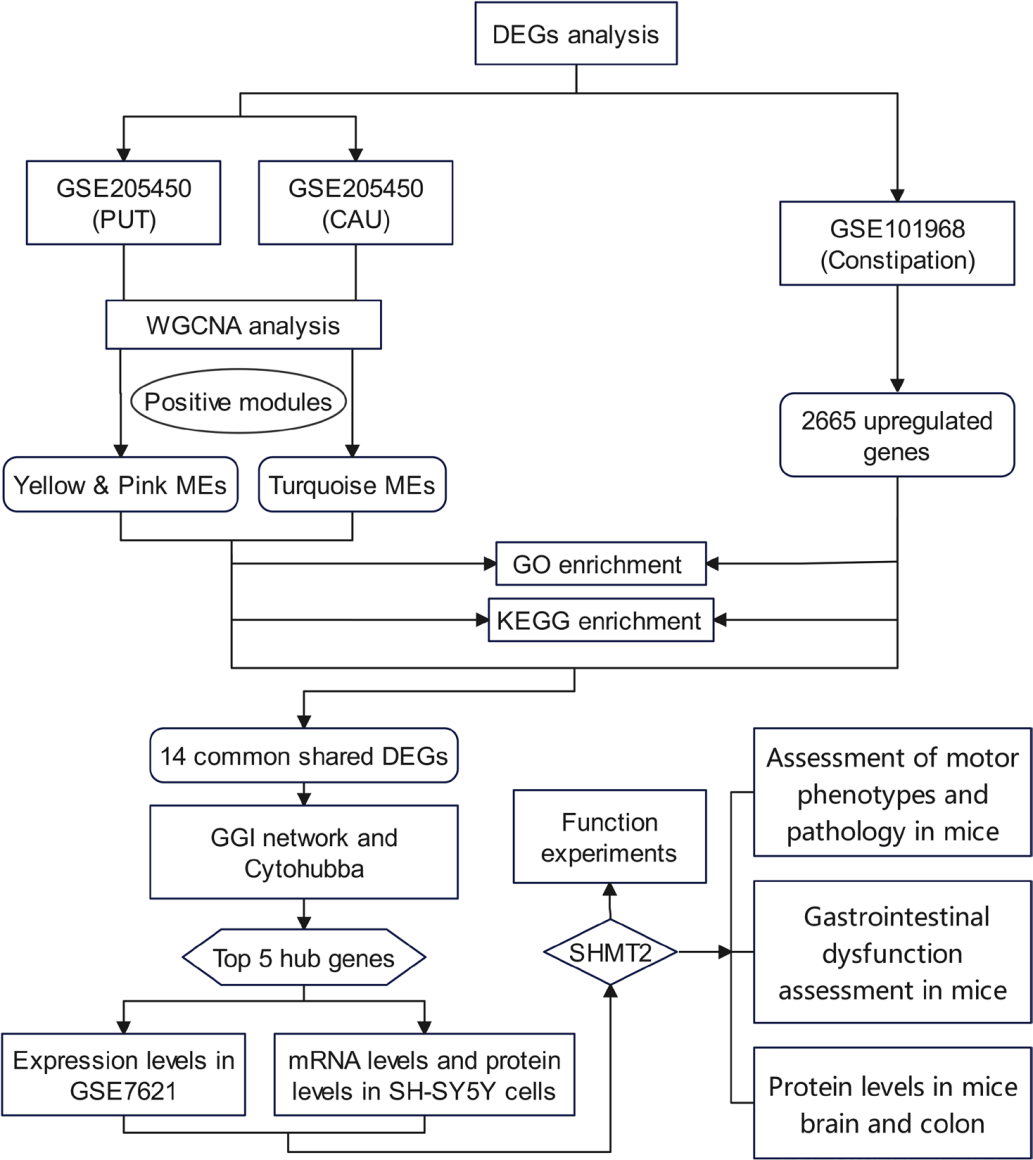
Study flowchart. This flowchart illustrates the analysis procedures utilized in this study. CytoHubba, a Cytoscape plugin for ranking nodes in a network by their network features; DEGs, differentially expressed genes; GEO, Gene Expression Omnibus; GGI, gene–gene interaction; GO, Gene Ontology; KEGG, Kyoto Encyclopedia of Genes and Genomes; MEs, module eigengenes; SHMT2, serine hydroxymethyltransferase 2; WGCNA, Weighted Gene Co‐expression Network Analysis.

The workflow of this study is illustrated in Figure 1, which provides an overview of the study design and analysis procedures. This comprehensive approach allowed us to investigate the molecular mechanisms linking Parkinson's disease and constipation.

### 2.1 Data Collection

For PD, we included 57 PD samples and 73 healthy control samples from GSE205450, as well as 16 PD samples and 9 healthy control samples from GSE7621. For constipation, the GSE101968 dataset, containing 3 disease and 3 normal samples, was selected for analysis. Detailed descriptive information of datasets was shown in Table 1. All datasets were obtained from the publicly accessible database GEO (Gene Expression Omnibus, www.ncbi.nlm.nih.gov/geo/).

**TABLE 1:** Basic information of GEO datasets used in the study.

GEO series	Data type	Platform	Sample description and size (*n*)	Tissue
GSE205450	Discovery set	GPL24676	PD patients (57) Caudate samples: total vs. disease duration info (28:25) Putamen samples: total vs. disease duration info (29:27) Healthy controls (73)	Striatum
GSE101968	Discovery set	GPL16791	Constipated patients (3) Healthy controls (3)	Rectal wall
GSE7621	Validation set	GPL570	PD patients (16) Healthy control (9)	Substantia nigra

Abbreviations: GEO, Gene Expression Omnibus; GPL, Gene Expression Omnibus Platform; GSE, Gene Expression Omnibus Series; PD, Parkinson's disease.

### 2.2 Identification of Differentially Expressed Genes (DEGs) and Hub Modules by WGCNA

DEGs were meticulously analyzed using the R limma package across all three datasets. DEGs were characterized by having a false discovery rate (FDR) < 0.05 and |Log2FC| ≥ 0.1 [11]. This threshold was selected to comprehensively identify potential DEGs, particularly those that may act synergistically in biological pathways, thereby enhancing the reliability of our findings. Subsequently, these DEGs were employed for further analysis aimed at identifying genes with altered expression between disease and normal samples in the test datasets, in addition to facilitating hub gene validation in GSE7621. A weighted gene co‐expression network analysis (WGCNA) was conducted using PD samples from the GSE205450 dataset, concentrating on samples with accessible disease duration information, and implementing a cutoff of 20 years. In short, we calculated all possible Pearson correlation matrices and then used the “picksoftThreshold” function to convert them into an adjacency matrix with soft threshold capability. The dynamic tree cutting algorithm was utilized to identify highly interconnected genes. The minimum module size was established based on the specific characteristics of each DEG group, with a default threshold for module merging set at 0.25. Each module was assigned a unique color and contained specific genes. Ultimately, distinct module eigengenes (MEs) were derived based on the first principal component of module expression. The relationships between module eigengenes and traits were meticulously evaluated by examining the associations between MEs and disease duration (less than 20 years vs. 20 years or more) to identify statistically significant associations. The modules exhibiting the most pronounced positive correlations from PD caudate and putamen samples were selected for subsequent in‐depth analysis.

### 2.3 Gene Ontology (GO) and Pathway Enrichment Analysis

The R software package “clusterprofiler” was used for Gene Ontology (GO) and Kyoto Encyclopedia of Genes and Genomes (KEGG) enrichment analyses. GO terms and KEGG pathways with an adjusted *p*‐value of less than 0.05 were deemed statistically significant. The results of these analyses were visualized using the ggplot2 R package.

### 2.4 Construction of Gene–Gene Interaction (GGI) Networks and Identification of Shared Hub Biomarkers

Co‐expressed DEGs within the most prominent positive modules derived from PD caudate and putamen samples, along with all upregulated DEGs from the constipation (GSE101968) dataset, were identified via intersection analysis. A GGI network of all candidate co‐expressed genes was constructed using the GeneMinia (http://www.genemania.org) database [12]. Cytoscape software was employed to identify key genes and to generate a visual representation of the network. The top 10 genes within the GGI network were identified through the application of the maximal clique centrality (MCC) algorithm in Cytoscape, a method specifically designed for the identification of hub genes and sub‐networks from complex interactomes.

### 2.5 Cell Culture, Transfections, and PFFs Production

Human SH‐SY5Y cells obtained from Procell Life Science and Technology Co. Ltd. (CL‐0208, Wuhan, China) were cultured in MEM/F12 medium supplemented with 10% fetal bovine serum (FBS) at 37°C under 5% CO_2_. To induce a neuronal phenotype, the cells were subjected to treatment with 10 μM retinoic acid (RA) for a duration of 72 h, followed by an additional treatment with 80 nM 12‐O‐tetradecanoylphorbol‐13‐acetate (TPA) for 72 h. Recombinant human A53T mutant α‐syn monomer solution from Genemei Biotech (Guangzhou, China) was prepared to a final concentration of 3 mg/mL (~100 μM) with a purity of 98% and stored at −80°C. For transfections, SHMT2 siRNA (50 nM, Haixing Biosciences, China; see Table S2 for sequences) was transfected into neuronally differentiated SH‐SY5Y cells using the jetPRIME transfection reagent, followed by incubation at 37°C for 48 h. The α‐syn monomer solution was dissolved in PBS and incubated in a rotary shaker at 150 rpm for 72 h at 37°C to generate α‐synuclein preformed fibrils (PFFs). In the α‐syn group, SH‐SY5Y cells were subjected to treatment with preformed fibrils (PFFs) at a concentration of 25 μM for a duration of 24 h [13], whereas the control group received treatment with PBS.

### 2.6 qRT‐PCR Validation of the Hubgenes

Total RNA was extracted from cultured cells using Trizol reagent (Servicebio, China, Cat G3013). A Prime Script RT kit (Servicebio, China, Cat G3337) was used to convert 1 μg of RNA into complementary DNA (cDNA) for quantitative PCR (qPCR). SYBR Premix Ex Taq (Servicebio, China, Cat. G3326) was utilized to quantify mRNA levels. Relative mRNA expression levels were quantified employing the $2^{-\Delta \Delta C_{t}}$ method. The primer sequences employed for qPCR analysis are detailed in Table S1.

### 2.7 Western Blot Analysis

Cells were lysed using RIPA buffer containing a protease inhibitor cocktail (Cwbiotech, China, Cat. CW2200S). Protein concentration was measured using the BCA assay (Cwbiotech, China, Cat. CW0014S). A total of 20 μg protein per lane was loaded onto a 12% SDS‐PAGE gel. The resolved proteins were subsequently transferred to a PVDF membrane (Millipore, USA). The membrane was blocked with a solution of 5% skim milk to prevent nonspecific binding. Subsequently, the membrane was incubated overnight at 4°C with primary antibodies, including Anti α‐syn (#45083, CST), Anti‐SHMT2 (#ab316328, Abcam), Anti‐DAT (#ab184451, Abcam), Anti‐Vinculin (#13901, CST), Anti‐cleaved caspase‐3 (#AF7022, Affinity), and GAPDH (#AF7021, Affinity). Following this, the membrane was incubated for 1 h at room temperature with HRP‐conjugated secondary antibodies. Signals were detected using a Chemiluminescence ECL kit (EpiZyme, China, Cat SQ201). A chemiluminescence imaging system was employed for analysis.

### 2.8 Detection of JC‐10 and ROS in Cellular Models

In the SH‐SY5Y cellular model, mitochondrial membrane potential was assessed using the JC‐10 assay. JC‐10 dye was prepared by diluting JC‐10 (500×) with 1× JC‐10 staining buffer. Cells were incubated with the JC‐10 working solution for 30 min at 37°C, then observed under a fluorescence microscope with excitation/emission wavelengths of 490/525 nm (monomer, green) and 525/590 nm (aggregate, red). For ROS detection, cells were loaded with DCFH‐DA working solution for 30 min at 37°C. Fluorescence was measured at 488/525 nm, with intensity reflecting intracellular ROS levels. Both assays were conducted following the manufacturer's protocols (Wuhan Service bio Biotechnology Co. Ltd).

### 2.9 Animal Model and Behavior Test

Eight‐week‐old female C57BL/6 mice were obtained from the Laboratory Animal Center of Sun Yat‐Sen University (Guangzhou, China). A53T transgenic mice were obtained from GemPharmatech Co. Ltd. All experimental procedures were conducted in compliance with the National Institutes of Health Guide for the Care and Use of Laboratory Animals (8th edition, 2011). The mice were housed in an air‐conditioned room maintained at 23°C ± 1°C and 55% ± 5% relative humidity. They were provided standard food and tap water ad libitum under a 12‐h light/dark cycle (lights on from 8:00 a.m. to 8:00 p.m.). All experimental procedures were designed to minimize the number of animals used and to reduce their suffering. The study received approval and oversight from Sun Yat‐Sen University (Approval number: SYSU‐IACUC‐2024‐002260).

The total experimental period lasted 27 weeks. Following 1 week of acclimatization, the mice were randomly allocated into three experimental groups (*n* = 5 per group) based on body weight: PFF‐induced models (PFF), sham controls (SHAM), and loperamide‐induced models (LOP). The PFF mouse model was established as delineated previously [14]. In brief, the mice were anesthetized using 0.3% sodium pentobarbital (30 mg/kg, i.p.) followed by stereotaxic injection of 10 μg PFFs (AP: 0.2 mm, ML: −2.0 mm, DV: 2.6 mm). The sham operation control group underwent the same operation, but was injected with 0.9% normal saline. At the 24th week, the LOP group was gavaged with loperamide hydrochloride suspension (10 mg/kg) twice daily (9:00 a.m. and 3:00 p.m.) for 2 weeks to induce a functional constipation model [15]. Previous literature [16] indicates that A53T transgenic mice exhibit motor and gastrointestinal symptoms by 3 months of age. In this study, motor and gastrointestinal functions in A53T mice were evaluated every other month, and tissue samples were collected at 3 months of age for further analysis.

To assess motor symptoms, mice were subjected to a series of behavioral tests, including the rotarod test, pole test, and grid hanging test, to evaluate motor coordination, muscle rigidity, and grip strength. These tests were administered before treatment and at the 5th, 14th, and 27th weeks. The animals underwent a training regimen for three consecutive days prior to the initial testing session, or a single day of refresher training if they had been previously tested [17, 18, 19]. Statistical analyses were conducted based on the average time recorded from three trials of each test.

In the rotarod test, the mice were initially placed on the rotating rod for a duration of 30 s to acclimate, followed by training at a constant rotational speed of 10 rpm for 300 s. A machine equipped with automatic timers and a smooth linear acceleration process was used in this study (Jinan Yiyan Science and Technology Development Co. Ltd., China, Cat. YLS‐4D). During the formal test, mice were gently placed on a rotarod that accelerated from 4 to 40 rpm over 300 s, and the latency to fall was recorded.

In the pole test, a metal rod measuring 50 cm in length and 10 mm in diameter, wrapped with bandage gauze, was utilized. During the actual testing procedure, the time to turn and total time to place all four paws on the base were measured after placing head down on the top of the pole. The maximum cutoff time was 60 s [20].

The grid hanging test, also known as the cage top or four‐limb hanging test, measured muscular strength and endurance by placing mice on a wire grid, inverting it, and recording the time until they fell (maximum cutoff: 420 s). Prior to testing, the mice underwent three trials of 30 s each for training, and the grid was sanitized with 70% ethanol between trials to ensure consistency and reduce stress levels.

### 2.10 Assessment of Gastrointestinal Function

One day following the final administration of loperamide, at approximately the 27th week, all mice underwent comprehensive gastrointestinal function assessments, which corresponded to 3 months of age for the A53T mice. To facilitate the 1‐h stool assessment, the animals were subjected to overnight fasting. The following day, the mice were granted access to food for a duration of 2 h prior to testing. The cumulative weight and number of feces produced within 1 h were collected by relocating the mice to a novel environment at the same time of day (10–11 a.m.). Subsequently, the feces were dried at 65°C overnight, and the dry weight was subsequently quantified. The moisture content was calculated as a percentage derived from the difference between wet weight and dry weight. Carmine was used as a marker in the whole‐intestinal transit test, and 0.3 mL of a solution containing 3 g of carmine in 50 mL of 0.5% methylcellulose was administered orally to each mice. The time taken for the oral marker to appear in the feces was recorded. Fecal pellets were monitored every 10 min for the appearance of the first red pellet. Progress was monitored 6 h after the administration of the marker. In the upper gastrointestinal transit test [21], mice were administered a carmine solution 30 min prior to anesthesia. The total length of the intestinal canal (from the pylorus to the terminal rectum) and the distance traveled by the leading edge of the carmine marker paste were measured. Furthermore, the length of the colon was also measured. The propulsion rate of the carmine marker was calculated utilizing the following equation:

Intestinal propulsion rate (%) = (Migration distance of barium sulfate paste)/(Whole length of intestinal canal) × 100%.

### 2.11 Histological Analysis, Immunohistochemistry, and Immunofluorescence

Mice were anesthetized and transcardially perfused with cold PBS, followed by 4% paraformaldehyde (PFA) for tissue fixation. Colon and brain tissues were rinsed with PBS to remove residual blood and cellular debris. For histopathological evaluation, tissues were fixed overnight in 4% PFA and subsequently embedded in paraffin. Serial 5‐μm‐thick paraffin sections were prepared and subjected to hematoxylin and eosin (H&E) staining for morphological analysis. Following dewaxing, rehydration, and antigen retrieval, immunohistochemistry (IHC) and Immunofluorescence (IF) was conducted on colon and brain sections using established protocols [14]. Primary antibodies included Anti‐phosphor‐α‐syn (#ab51253, Abcam), Anti‐SHMT2 (#ab316328, Abcam), Anti‐HuD (#sc‐48,421, Santa Cruz), and Anti‐TH (#MAB318, Merck Millipore). Four representative images at 4×, magnification, each capturing distinct regions of the SN, were acquired and compiled into a composite figure. To evaluate nigrostriatal dopaminergic neuron terminals, tyrosine hydroxylase (TH) optical density was quantified using ImageJ software. For SHMT2 (Serine hydroxymethyltransferase 2) expression analysis, mean optical density was measured in colon tissues, while IHC scores for SHMT2 in brain sections were calculated by multiplying staining intensity (graded on a scale of 1–3) by the percentage of antibody‐positive cells [22]. Immunofluorescence double labeling experiments were performed for HuD with phosphor‐α‐syn and for HuD with SHMT2. Primary antibodies were incubated overnight at 4°C (anti‐HuD, 1:50; anti‐phosphor‐α‐syn, 1:500; anti‐SHMT2, 1:2500), followed by detection with Alexa Fluor 488 and 594 secondary antibodies (Invitrogen, 1:500) for 1 h at room temperature.

### 2.12 Statistical Analysis

Statistical analyses were performed using R (version 4.3.2) or GraphPad Prism 8. Quantification of immunohistochemistry, immunofluorescence, and Western blot data was performed using ImageJ, with results expressed as mean ± SEM. The Shapiro–Wilk test was used to assess data normality, and homogeneity of variances was tested using Bartlett's or Brown–Forsythe tests as appropriate. For two‐group comparisons, normally distributed data with equal variances were analyzed with an unpaired two‐tailed Student's *t*‐test; otherwise, the Mann–Whitney U test was used. For three or more independent groups, one‐way ANOVA with Tukey's post hoc test was applied if normality and homoscedasticity were confirmed. If these assumptions were violated, the nonparametric Kruskal–Wallis test with Dunn's post hoc test was performed. Behavioral score data were analyzed using two‐way repeated measures ANOVA, with treatment groups (Sham, PFF, LOP) as between‐subjects factors and time points (1, 3, and 6 months) as within‐subjects factors. Normality and homogeneity of variances were confirmed for behavioral scores and their change scores across groups, meeting the assumptions for repeated measures ANOVA. Significant main effects were followed up with Tukey's post hoc test to identify specific differences between time points or treatment groups. A two‐tailed *p* value ≤ 0.05 was considered statistically significant.

## 3 Results

### 3.1 Identification of DEGs

**FIGURE 2:**
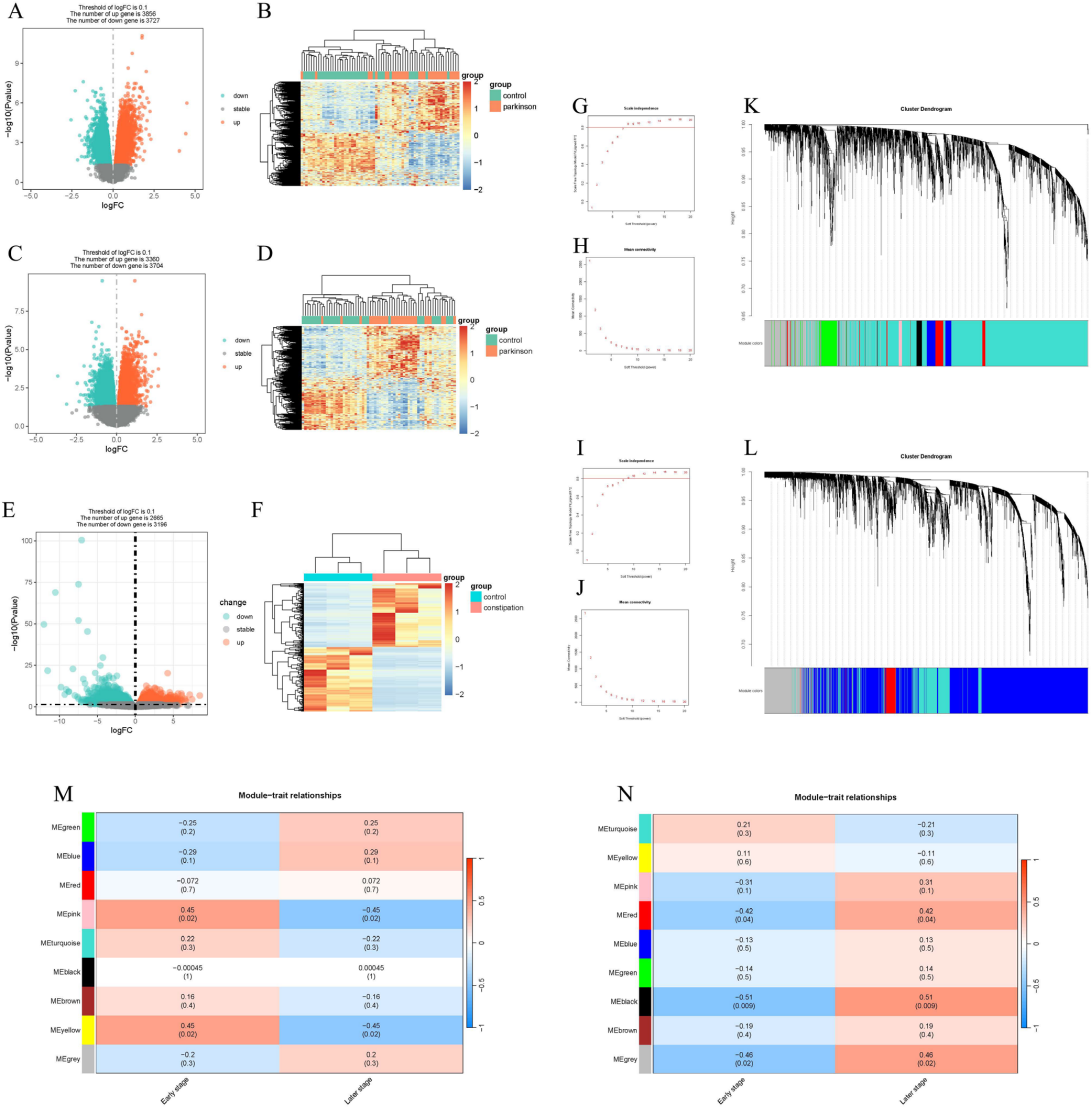
Analysis of DEGs and Hub Modules by WGCNA. (A, C, E) Volcano plots depicting the distribution patterns of all genes in the control and PD putamen (A), caudate (C), and GSE101968, highlighting significant DEGs. (B, D, F) Heatmaps comparing the expression of the top 50 DEGs between control and PD putamen samples (B), caudate samples (D), and constipation patients (F). (G, H) Analysis of the scale‐free fit index for various soft‐thresholding powers (*β*) and the mean connectivity for various soft‐thresholding powers in the PD putamen. (I, J) Analysis of the scale‐free fit index for various soft‐thresholding powers (*β*) and the mean connectivity for various soft‐thresholding powers in the PD caudate. (K, L) Dendrograms of all genes clustered based on a dissimilarity measure (1‐TOM) in the PD putamen (K) and caudate (L). (M, N) Heatmaps of module–trait relationships in the putamen (M) and caudate (N). DEGs, differentially expressed genes; ME, module eigengene; PD, Parkinson's disease.

We first identified DEGs between PD patients and healthy individuals to focus on genes differentially expressed in PD, which was crucial for narrowing down the analysis to those potentially involved in PD pathogenesis. In the PD discovery set, DEGs analysis identified 3360 upregulated and 3704 downregulated RNAs in the PD caudate compared to controls, as well as 3856 upregulated and 3727 downregulated RNAs in the PD putamen relative to controls. Heatmaps illustrating group‐wise expression patterns across all samples and corresponding volcano plots are presented in Figure 2A–D. Similarly, in the constipation discovery cohort, DEG analysis revealed 2665 upregulated and 3196 downregulated RNAs, as illustrated in Figure 2E,F.

### 3.2 Construction of the Co‐Expression Network Using WGCNA

The analysis of RNA‐seq data derived from the striatum of PD patients exhibiting varying disease durations not only facilitates the identification of gene modules associated with disease progression but also provides critical insights into the molecular mechanisms underpinning PD pathogenesis. To this end, we applied WGCNA to caudate and putamen samples with available disease duration data. To establish the optimal soft thresholding value (*β*) for constructing the co‐expression network, we systematically evaluated values ranging from 1 to 20 based on two key metrics: scale independence and mean connectivity. The results of these analyses confirmed that the resultant network conformed to a scale‐free topology, which is a prerequisite for conducting robust network analysis. The optimal *β* value was subsequently utilized to construct the co‐expression network, with *β* = 14 for putamen samples and *β* = 12 for caudate samples, as depicted in Figure 2G–J. By employing a dynamic tree cut‐off threshold of 0.25 for module merging, we successfully identified 11 co‐expression modules in the PD putamen and 9 modules in the PD caudate, utilizing similarity‐based clustering (Figure 2K,L).

Subsequently, Pearson correlation coefficients were computed to evaluate the relationships between MEs and sample traits. In the PD putamen, the yellow and pink module exhibited a significant positive correlation with early‐stage disease (*r* = 0.45), whereas the green (*r* = −0.25) and blue (*r* = −0.29) modules revealed pronounced negative correlations with early‐stage disease (Figure 2M). In the PD caudate, the turquoise module exhibited the highest positive correlation (*r* = 0.21), whereas the black module displayed a pronounced negative correlation (*r* = 0.51) (Figure 2N). Ultimately, we intersected the most positive correlation with early‐stage disease MEs identified by WGCNA in both PD putamen and caudate samples with upregulated DEGs from GSE101968, thereby identifying 14 shared DEGs between PD and constipation (Figure 4A). This intersection allowed us to narrow down DEGs that are potentially implicated in both diseases.

### 3.3 Functional Enrichment Analyses

**FIGURE 3:**
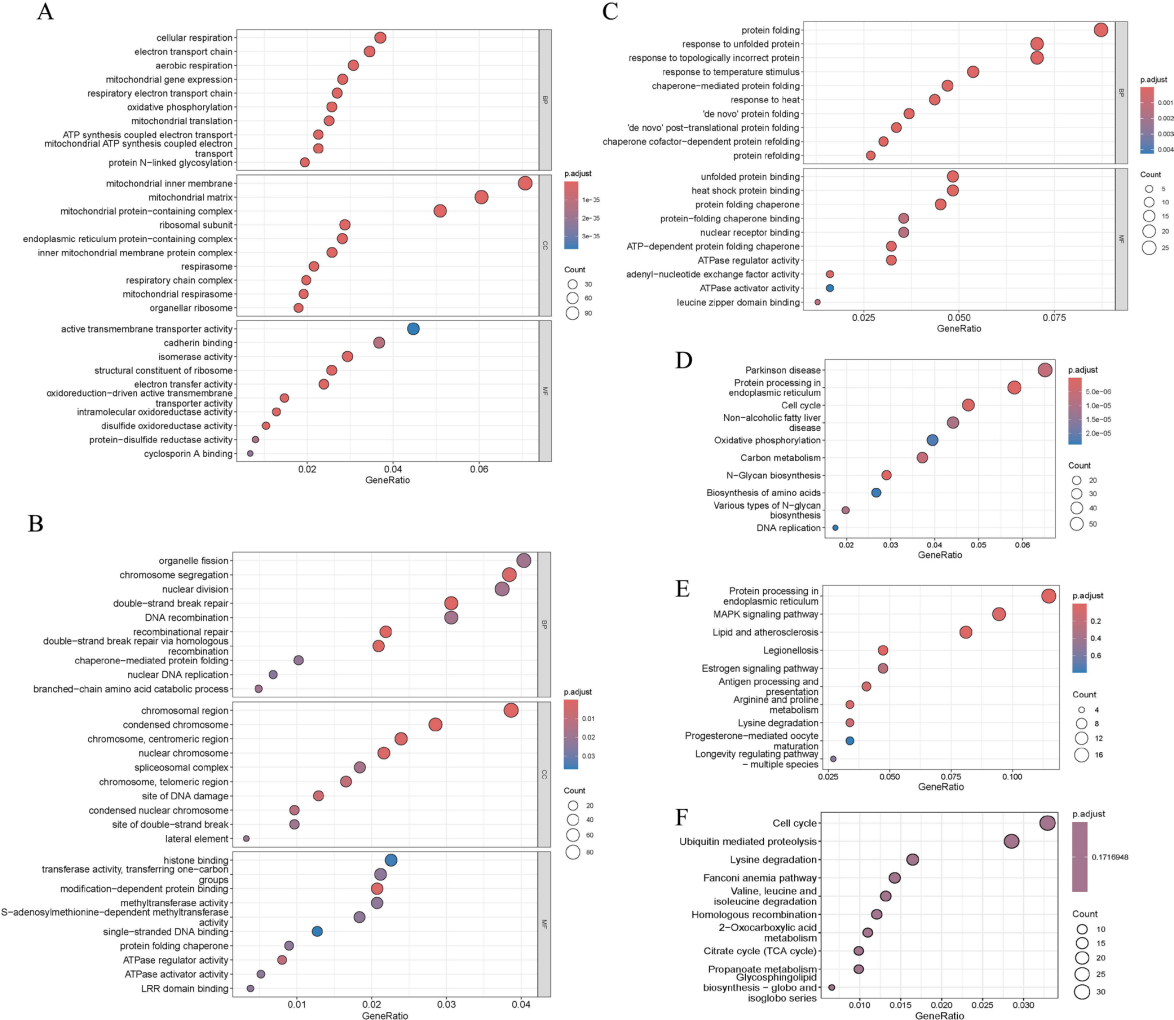
Functional enrichment based on selected DEGs. (A–C) Top 10 enrichment results of selected DEGs for GO terms in the caudate module (A), putamen modules (B), and GSE101968. (D–F) The top 10 KEGG pathway enrichment results of selected DEGs in GSE101968 (D), putamen modules (E), and caudate module (F). BP, biological process; CC, cellular component; DEGs, differentially expressed genes; GO, Gene Ontology; KEGG, Kyoto Encyclopedia of Genes and Genomes; MF, molecular function.

To investigate biological data of the selected DEGs in PD and constipation, the GO functional and KEGG pathway enrichment were conducted. The top GO analysis of the upregulated DEGs within the constipation datasets revealed a predominant involvement of these genes in energy metabolism and protein modification concerning the biological process (BP). Furthermore, in terms of cellular components (CC), these genes exhibited a crucial role in structural and functional contexts associated with mitochondria and the endoplasmic reticulum. Within the molecular function (MF) category, it was noted that these genes were chiefly engaged in processes such as protein folding, redox reactions, and transmembrane transport, thereby reflecting the underlying molecular mechanisms governing protein synthesis and energy metabolism within the cells (Figure 3A). As shown in Figure 3D, the DEGs that were upregulated in patients with constipation exhibited significant enrichment across various pathways, including DNA replication and cellular cycle, N‐glycan biosynthesis, endoplasmic reticulum processing, oxidative phosphorylation, as well as a specific pathway pertinent to PD.

Regarding the PD putamen, the DEGs identified within the yellow and pink module predominantly engage in protein folding and chaperone functions (Figure 3C). Concurrently, these DEGs also demonstrate substantial enrichment in pathways associated with protein processing, metabolic processes, signal transduction, and immune respond (Figure 3E). The top GO terms delineated the regulation of ontogeny and metabolic processes concerning branched‐chain amino acids, nuclear DNA replication, protein folding of molecular partners, as well as dual‐chain fracture repair exhibited significant alterations within the turquoise module in the PD caudate (Figure 3B). As illustrated in Figure 3F, the top KEGG pathways were identified as encompassing a diverse array of biological processes, which include metabolic pathways, DNA repair mechanisms, protein degradation, and regulation of the cell cycle. Although the DEGs within the turquoise module demonstrated enrichment across several KEGG pathways (Table S3), the *p*‐value following statistical correction exceeded 0.05, thereby indicating that such enrichments may not achieve statistical significance. The GO and KEGG enrichment analysis of 14 shared DEGs are presented in Table 2.

**TABLE 2:** Results from GO and KEGG enrichment analysis of shared DEGs.

ID	Ontology	Description	Gene ratio	Bg ratio	*p*.adjust
GO:0046655	BP	Folic acid metabolic process	2/13	15/18870	2.39E−02
GO:0046653	BP	Tetrahydrofolate metabolic process	2/13	16/18870	2.39E−02
GO:0006986	BP	Response to unfolded protein	3/13	137/18870	2.40E−02
GO:0006760	BP	Folic acid‐containing compound metabolic process	2/13	26/18870	2.40E−02
GO:0031468	BP	Nuclear membrane reassembly	2/13	26/18870	2.40E−02
GO:0035966	BP	Response to topologically incorrect protein	3/13	159/18870	2.40E−02
GO:0042558	BP	Pteridine‐containing compound metabolic process	2/13	32/18870	2.80E−02
GO:0006730	BP	One‐carbon metabolic process	2/13	40/18870	3.54E−02
GO:0006458	BP	‘De novo’ protein folding	2/13	41/18870	3.54E−02
GO:0071763	BP	Nuclear membrane organization	2/13	44/18870	3.54E−02
GO:0006457	BP	Protein folding	3/13	223/18870	3.54E−02
GO:0030850	BP	Prostate gland development	2/13	50/18870	4.01E−02
GO:0006998	BP	Nuclear envelope organization	2/13	54/18870	4.35E−02
GO:0006767	BP	Water‐soluble vitamin metabolic process	2/13	60/18870	4.44E−02
GO:0071709	BP	Membrane assembly	2/13	60/18870	4.44E−02
GO:0140014	BP	Mitotic nuclear division	3/13	274/18870	4.44E−02
GO:0044091	BP	Membrane biogenesis	2/13	64/18870	4.57E−02
GO:0001655	BP	Urogenital system development	2/13	67/18870	4.57E−02
GO:0006919	BP	Activation of cysteine‐type endopeptidase activity involved in apoptotic process	2/13	68/18870	4.57E−02
GO:0002711	BP	Positive regulation of t cell mediated immunity	2/13	69/18870	4.57E−02
GO:0005759	CC	Mitochondrial matrix	4/13	487/19886	1.30E−02
hsa00670	Metabolism	One carbon pool by folate	2/9	39/8541	1.58E−02

Abbreviations: BP, biological processes; CC, cellular components; DEGs, differentially expressed genes; GO, Gene Ontology; KEGG, Kyoto Encyclopedia of Genes and Genomes; MF, molecular functions.

### 3.4 Identification of Hubgenes From GGI Network Analysis

**FIGURE 4:**
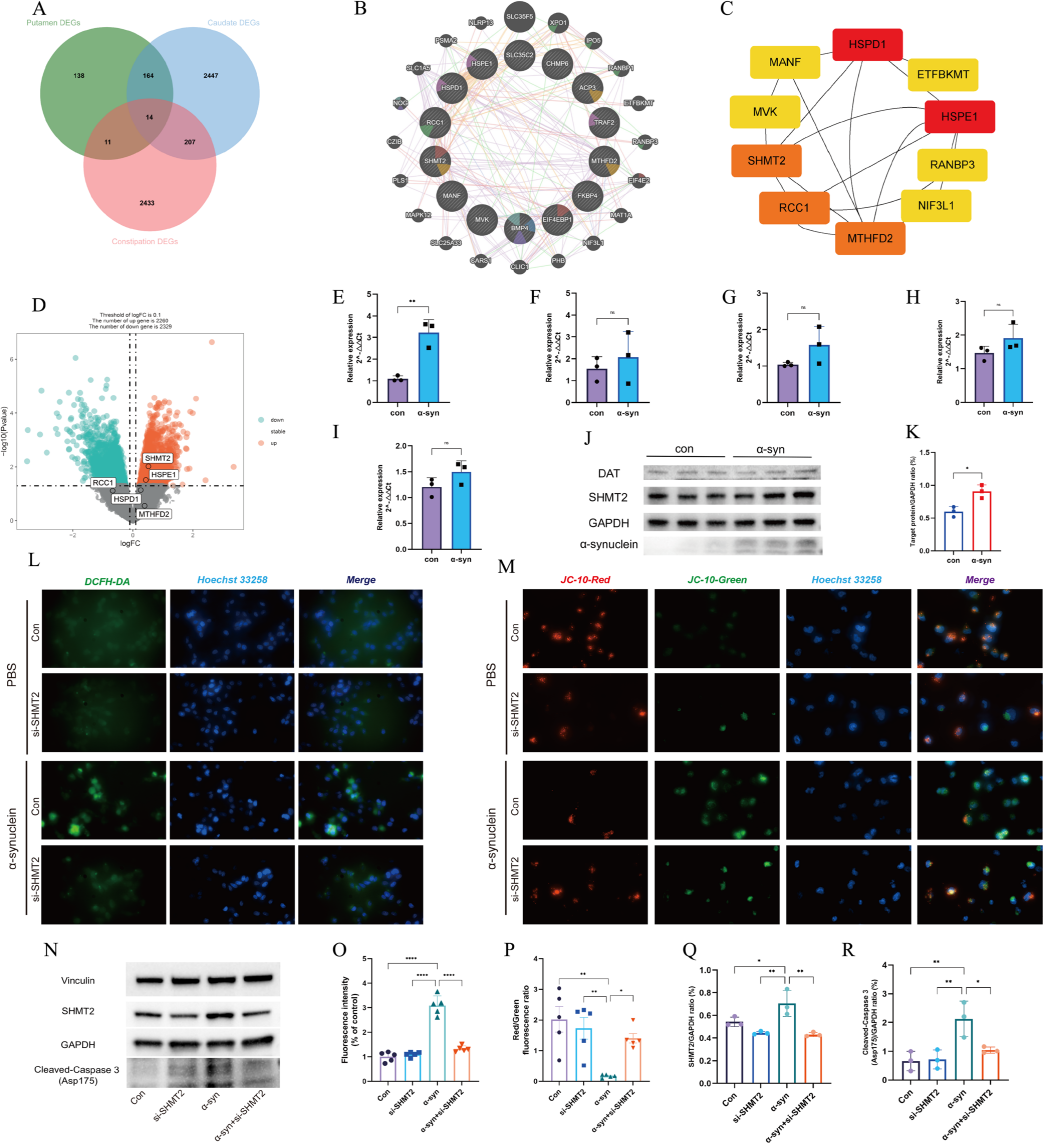
Identification and validation of hub genes in vitro. (A) A Venn diagram showing the overlapping genes between upregulated DEGs from GSE101968 and key modules identified by WGCNA in both putamen and caudate samples. (B) GGI network diagram of core genes. (C) Genes were clustered using cytoHubba. In the network, nodes represent genes, and edges indicate interactions between proteins. The color of each node reflects the strength of protein association, with red indicating a high level of association and yellow indicating a lower level. (D) A volcano plot depicting the distribution pattern of all genes in GSE7621; the top five hub genes are highlighted. (E–I) Expression levels of mRNA of SHMT2 (E), MTHFD2 (F), HSPD1 (G), HSPE1 (H), and RCC1 (I) in α‐syn group compare to control groups. Data were analyzed by qRT‐PCR. (J) Representative Western blot images show the expression of DAT, SHMT2, and α‐syn in SH‐SY5Y cells at 24 h after treatment with PFFs. (K) Graphical representation of the effect of exposed to 24 h α‐syn on the relative expression of SHMT2 protein level in SH‐SY5Y cells. (L) Representative images of the detection of ROS levels using a DCFH probe (green) and nuclei stained with DAPI (blue). (M) Representative images of JC‐10 staining. (N) Representative Western blot images showing Vinculin, SHMT2, GAPDH, and cleaved caspase‐3 expression in SH‐SY5Y cells under untreated, siRNA‐transfected, PFFs‐treated, and siRNA‐transfected + PFFs groups, *n* = 3 independent assays in duplicate. (O, P) The quantification of the DCFH‐DA intensity and red/green fluorescence intensity ratio. *n* = 5 independent assays in duplicate. (Q, R) Relative protein levels of SHMT2 (Q) and cleaved caspase‐3 (R) in SH‐SY5Y cells across untreated, siRNA‐transfected, PFFs‐treated, and siRNA + PFFs groups, assessed by immunoblot. *n* = 3 independent assays in duplicate. Data are presented as mean ± SEM. Statistical significance was determined using a two‐tailed unpaired Student's *t*‐test for (E–I, K), One‐way analysis of variance (ANOVA) was applied for (O–R). α‐syn, α‐synuclein; DAT, dopamine transporter; DEGs, differentially expressed genes; PFFs, α‐synuclein preformed fibrils; SHMT2, serine hydroxymethyltransferase 2; WGCNA, Weighted Gene Co‐expression Network Analysis.

To facilitate a more in‐depth understanding of the complex interactions among the 14 overlapping genes, a gene–gene interaction (GGI) network was carefully constructed utilizing the GeneMinia database, with the resultant network topology illustrated in Figure 4B. Furthermore, the CytoHubba plugin, embedded within Cytoscape version 3.8.2, was utilized to discern the most critical modules within the GGI network, thereby enabling the identification of key regulatory elements. The ensuing analysis revealed the presence of stable core clusters, consisting of 10 densely interconnected nodes, whose identification was facilitated by the MCC values, as visually represented in Figure 4C. Following the analysis, five top hub genes, namely HSPD1, HSPE1, RCC1, MTHFD2, and SHMT2, were selected for subsequent validation and verification as potential biomarkers, owing to their highest scores in the MCC values.

### 3.5 Validation and Confirmation of the Expression of Candidate Biomarkers In Vitro

The GSE7621 dataset, which includes both healthy controls and PD substantia nigra samples, was utilized to validate and further elucidate potential biomarkers among the top five shared genes. As illustrated in Figure 4D, the expression levels of RNAs for SHMT2 and HSPE1 were found to be exclusively upregulated in patients diagnosed with PD. Moreover, qRT‐PCR analysis revealed that only SHMT2 was significantly over‐expressed in differentiated SH‐SY5Y cell lines exposed to PFFs compared to the control group, whereas the expression levels of the other RNAs displayed no statistically significant differences (Figure 4E–I). To validate the expression of the candidate biomarker, the SH‐SY5Y cell line was utilized, which demonstrated the expression of the dopamine transporter (DAT) and effectively recapitulated dopaminergic‐like neurons in vitro following differentiation [23]. Additionally, the levels of SHMT2 were significantly observed to increase in response to PFF‐induced stimulation, while the levels of α‐syn showed notable elevation following treatment with PFFs, as depicted in Figure 4J,K.

We next sought to assess whether SHMT2, which correlates with neurodegenerative defects in PD models, serves merely as a marker or acts as a driver of the neurological changes observed under PFFs exposure. Our experiments on SH‐SY5Y cells demonstrated that SHMT2 siRNA treatment effectively reduced SHMT2 levels (Figure 4N), mitigated PFFs‐induced apoptosis by decreasing cleaved caspase‐3 and enhancing cell viability. Additionally, SHMT2 siRNA decreased ROS generation (Figure 4L), as measured by DCFH‐DA fluorescence, and preserved mitochondrial membrane potential (Figure 4M), indicated by JC‐10 staining.

### 3.6 Validation of the Expressions of Candidate Biomarkers In Vivo

#### 3.6.1 Animal Model and Behavior Test

**FIGURE 5:**
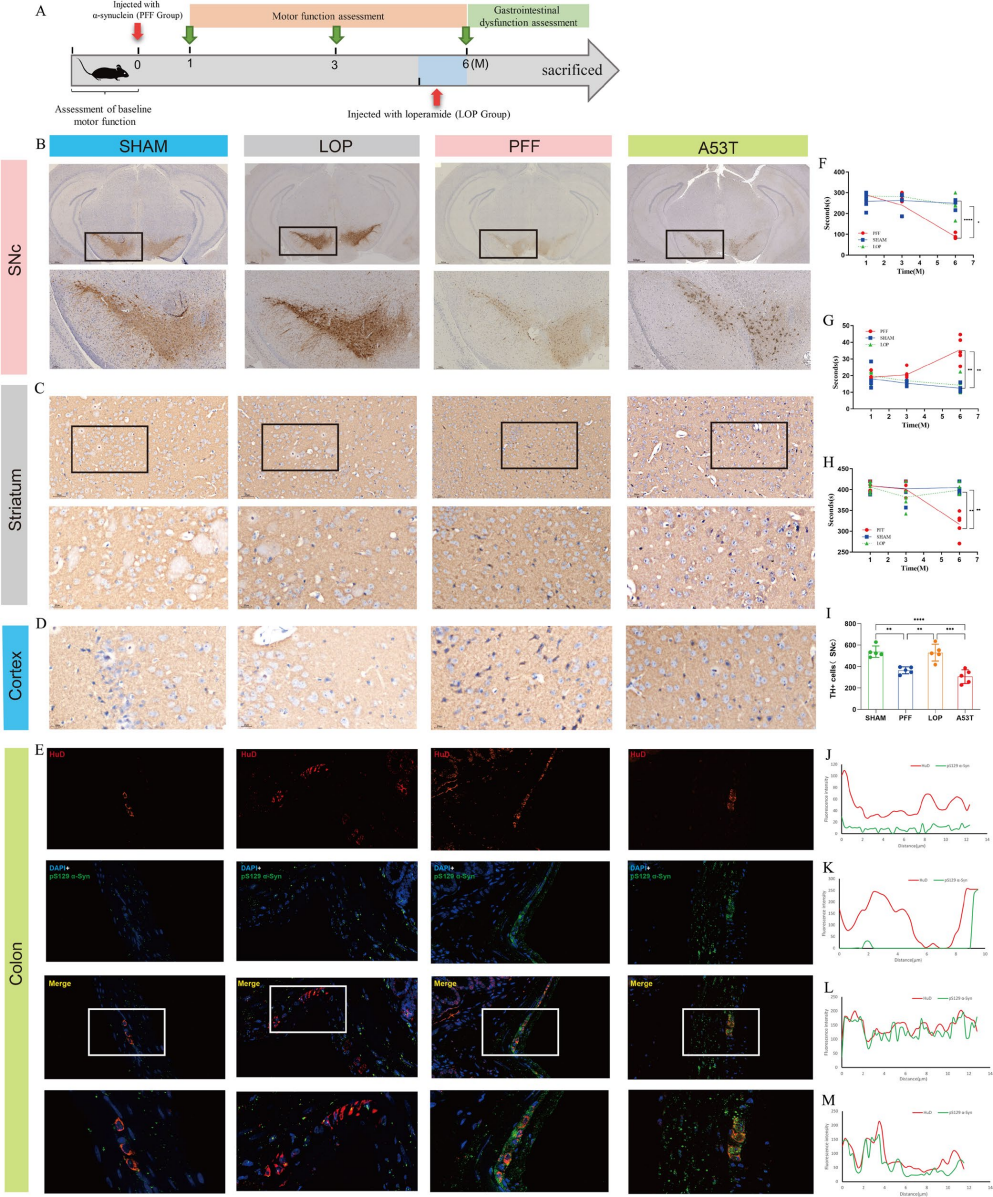
Motor function, dopamine neuron degeneration and α‐syn pathology assessments in mice. (A) Experimental workflow and timeline of treatments, behavioral experiments, and gastrointestinal function tests in SHAM, LOP, and PFF groups. (B) Representative IHC staining for TH in the SN of mice. Scale bars, 500 μm in 4×, and 100 μm in 20×. (C, D) Representative IHC staining for phosphor‐α‐syn in the stiatum (C) and cortex (D) of mice. Scale bars, 50 μm in 100× and 20 μm in 400×. (E) Representative images of phosphor‐α‐syn immunofluorescence (green) show colocalization with the HuD marker (red) in PFF and A53T mouse models, with increased expression compared to SHAM and LOP groups. Nuclei are labeled with DAPI. (F–H) Behavior tests involved in motor coordination and muscular strength assessed using rotarod test (F), pole test (G), and grid hanging test (H) over the experimental period in SHAM, LOP and PFF group (*n* = 5 independent assays in duplicate). (I) Graphs showing estimation of TH+ cells remaining in the SNpc (*n* = 5 independent assays in duplicate). (J–M) Immunofluorescence colocalization analysis of HuD (red) and pS129 α‐syn (green) in SHAM (J), LOP (K), PFF (L) groups, and A53T mouse model (M). *n* = 5 independent assays in duplicate. Fluorescence intensity profiles show the colocalization of pS129 α‐syn with the neuronal marker HuD, indicating the presence of phosphorylated α‐synuclein in neuronal structures. The *x*‐axis represents the distance along the analyzed line (μm), and the *y*‐axis represents the fluorescence intensity. This analysis demonstrates the colocalization of pS129 α‐syn with HuD‐positive neurons in PFF‐injected and A53T mouse models. Values are presented as mean ± SEM. One‐way ANOVA was applied when only one independent variable was considered (I). For evaluating the main effects of each variable (F‐H), two‐way ANOVA was used. Tukey's multiple comparisons test was employed to adjust the significance levels. (*p < 0.05, **p < 0.01, ***p < 0.001, ****p < 0.0001; *N* = 5 per group). α‐syn, α‐synuclein; IHC, immunohistochemistry; LOP, loperamide‐induced models; PFF, PFF‐induced models; pS129 α‐syn, phosphorylated α‐synuclein at Serine 129; SHAM, sham controls; SN, substantia nigra; SNc, substantia nigra pars compacta; TH, tyrosine hydroxylase.

The α‐syn PFF model not only faithfully recapitulates the motor symptoms of idiopathic PD but also encompasses a range of non‐motor symptoms, including cognitive and emotional impairments as well as autonomic dysregulation [24], thereby providing a comprehensive platform for elucidating the pathophysiology of PD and assessing prospective therapeutic interventions. To further validate the effectiveness of this model in evaluating motor function, we conducted a series of behavioral tests. To evaluate motor performance, mice were subjected to the rotarod test, pole test, and grid hanging test at 1, 3, and 6 months following PFFs or PBS injection, as shown in Figure 5A. In all assessed tests, the motor performance of the PFF group did not significantly differ from that of the sham and LOP groups at the 1‐ and 3‐month; however, a trend toward prolonged performance duration was observed in the rotarod and pole tests. A significant decline in performance was observed in the PFF group mice during the rotarod, pole, and grid hanging tests conducted at the 6‐month post‐injection (*p* < 0.01). In contrast, the sham and LOP group mice exhibited a nonsignificant trend toward performance deterioration over time. Collectively, these findings suggest that the PFF model effectively captures the progressive motor deficits that are characteristic of PD.

Complementary analysis of the dopaminergic system quantified TH immunoreactivity in four randomly selected representative micrographs per sample. Specimens included: (i) A53T transgenic mice at 3 months of age, and (ii) SHAM, LOP, and PFF group at 6 months post‐surgery, as visualized in Figure 5B. Representative images illustrate that PFFs‐injected mice and A53T transgenic mice exhibited a significantly lower number of TH‐positive cells compared to control mice, as shown in Figure 5I. This finding indicates a reduction in dopaminergic neuron density in the PFF‐injected group and A53T transgenic mice, consistent with the motor deficits observed. Furthermore, the presence of phosphorylated α‐syn was observed in the striatum and cortical regions of mice administered with PFFs injections and transgenic mice, while no significant accumulations were detected in the control and LOP groups, as shown in Figure 5C,D. This finding supports the hypothesis that PFF‐induced pathology contributes to the accumulation of phosphorylated α‐synuclein, which is a critical pathological feature of PD.

To determine whether PD‐associated colonic pathology involves the ENS, dual‐immunofluorescence colocalization analysis of HuD and p‐α‐syn was conducted. Notably, p‐α‐syn immunoreactivity colocalized with HuD within the ENS (Figure 5F–M) in both PFF‐injected and A53T transgenic mice. Conversely, control and LOP groups exhibited negligible p‐α‐syn/HuD colocalization.

#### 3.6.2 Assessment of Gastrointestinal Function

**FIGURE 6:**
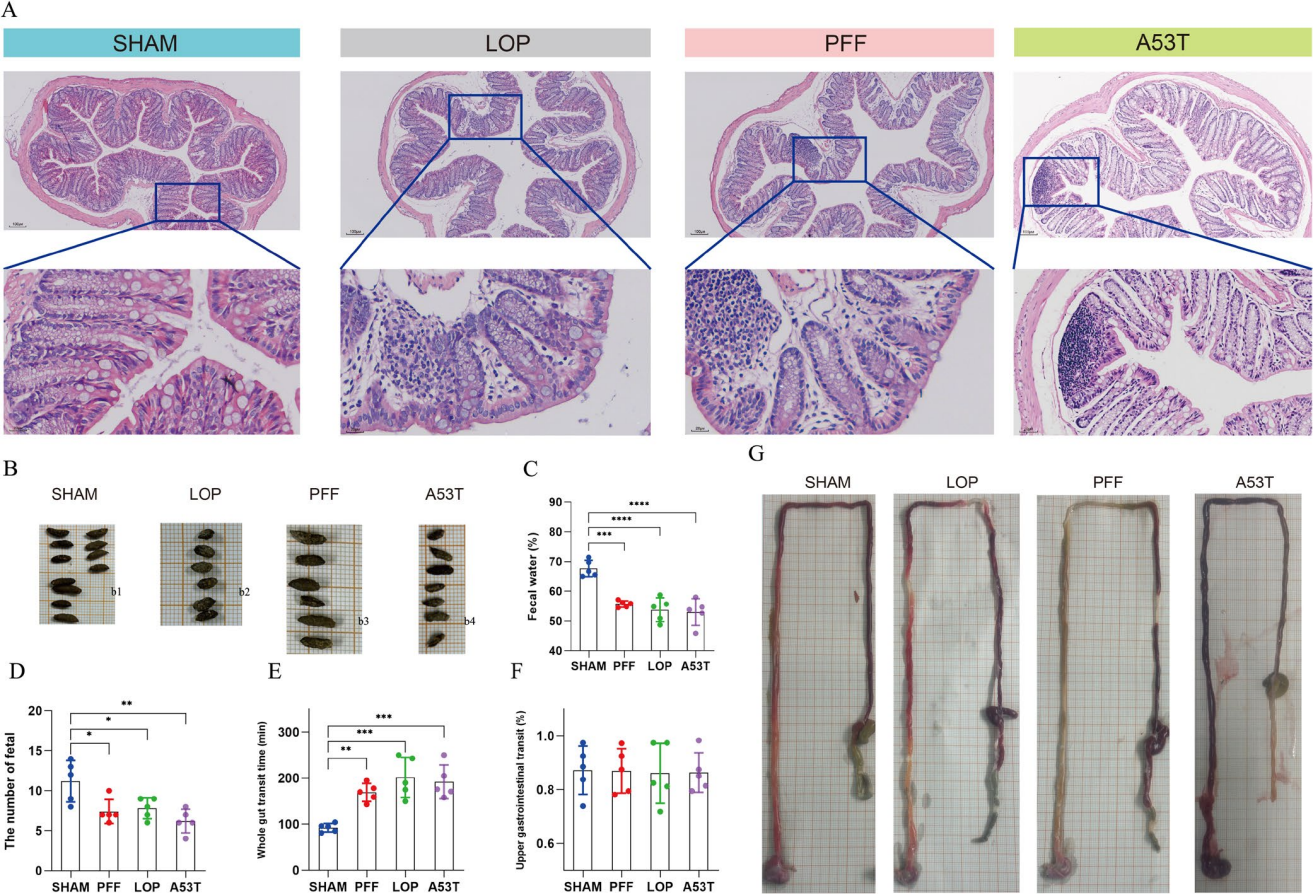
Gastrointestinal function assessments in mice. (A) Representative histological analysis of H&E staining of the colon tissues in mice (20×, scale bar = 100 μm; 400×, scale bar = 20 μm). (B) Representative images of stools collected in 1‐h trial from sham group (b1), LOP group (b2), PFF group (d3) and A53T transgenic mice. (C–F) Graphs showing the comparison of fecal water content (C), the number of fetal (D), whole gut transit time (E), and upper gut transit propulsion rate (F) among the three groups (*n* = 5 independent assays in duplicate). (G) Representative images showing the upper gut transit propulsion in Sham group (g1), LOP group (g2), PFF group (g3), and A53T model. All data are shown as mean ± SEM. One‐way analysis of variance (ANOVA) was applied for (C–F) Tukey's multiple comparisons test was employed to adjust the significance levels. (*p < 0.05, **p < 0.01, ***p < 0.001). H&E, hematoxylin and eosin; LOP, loperamide‐induced models; PFF, PFF‐induced models; SHAM, sham‐injected.

The structural integrity of the colon was preserved in the SHAM group, with no obvious pathological changes observed. In contrast, the LOP group exhibited mild to moderate tissue damage and inflammation, indicative of a moderate pathological response. Both PFF‐injected and A53T transgenic groups exhibited comparable levels of tissue destruction and inflammatory infiltration to the LOP group, consistent with the pathological hallmarks of the constipation model (Figure 6A).

Fecal specimens from PFF‐injected, LOP, and A53T transgenic groups exhibited desiccated, hardened, pelletized morphology with reduced surface reflectivity relative to controls (Figure 6B). Quantitatively, these groups demonstrated significantly decreased fecal moisture content and output frequency versus SHAM controls (PFF vs. SHAM: *p* < 0.01; LOP vs. SHAM: *p* < 0.05; A53T vs. SHAM: *p* < 0.05).

Whole‐gut transit time was significantly prolonged in loperamide‐induced, PFF‐injected, and A53T transgenic mice versus SHAM controls (*p* < 0.001, *p* < 0.01 and *p* < 0.001, respectively; Figure 6E), confirming profound gastrointestinal motility inhibition. To localize gastrointestinal dysfunction, upper GI transit was assessed pre‐euthanasia. Notably, no intergroup differences reached statistical significance in these measurements (Figure 6F).

#### 3.6.3 Validation of Biomarker Expressions Through Immunohistochemistry and Immunofluorescence

**FIGURE 7:**
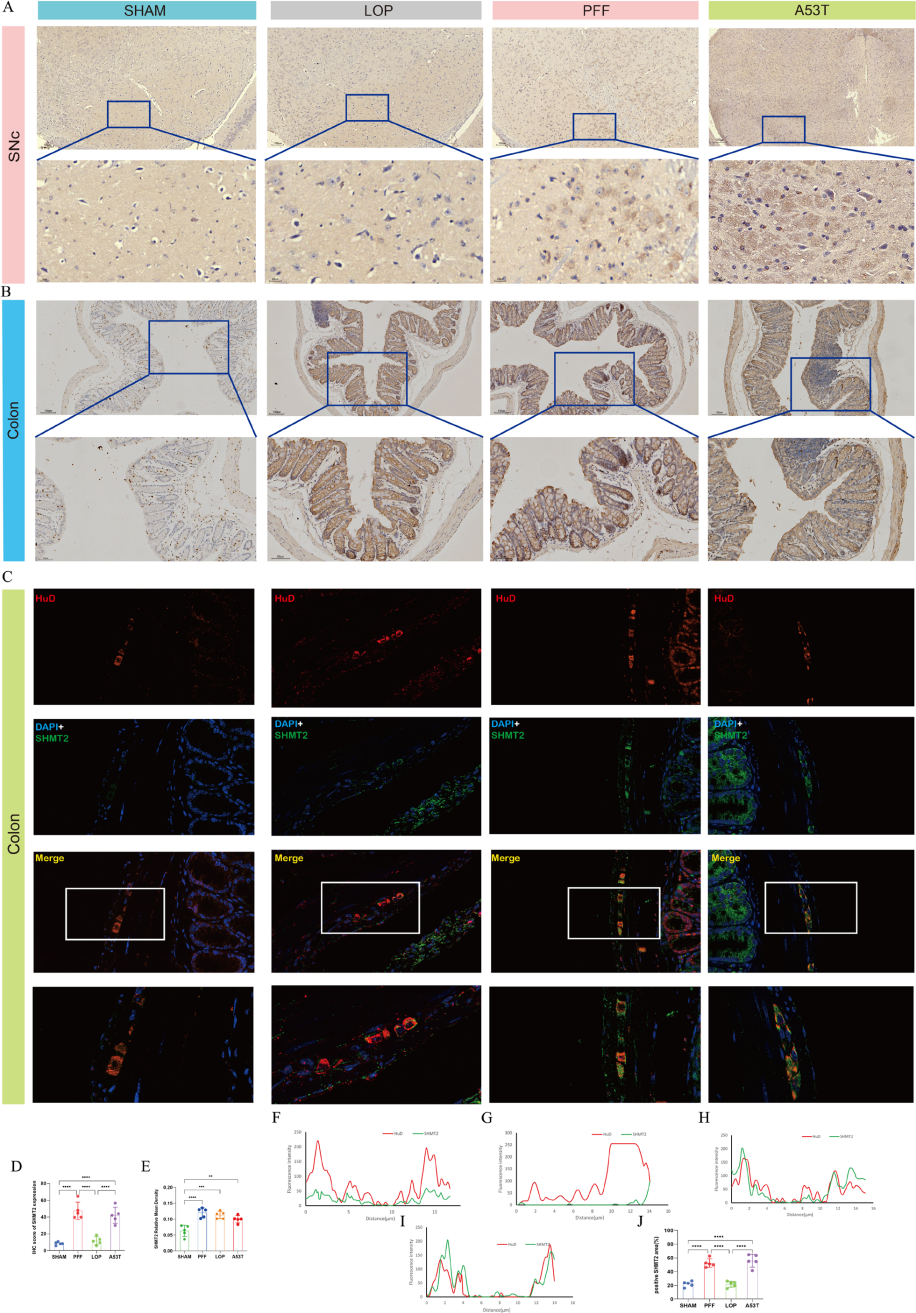
Analysis of SHMT2 protein expression levels in mice. (A) Representative histological analysis of IHC staining of SHMT2 in the substantia nigra tissue of mice (20×, scale bar = 100 μm; 400×, scale bar = 20 μm). (B) Representative IHC staining of SHMT2 in the colon tissue of mice (20×, scale bar = 100 μm; 400×, scale bar = 20 μm). (C) Representative immunofluorescence images show SHMT2 (green) colocalizing with the neuronal marker HuD (red) in the colon. In PFF‐injected and A53T mouse models, SHMT2 positively colocalizes with HuD, indicating elevated SHMT2 expression in these groups. Nuclei are stained with DAPI (blue). (D, E) Comparison of the mean IHC score in the midbrain (D) and relative mean density in the colon (E) of SHMT2 expression among the four groups. (F–I) Fluorescence intensity profiles show the colocalization of SHMT2 with the neuronal marker HuD. The *x*‐axis represents the distance along the analyzed line (μm), and the *y*‐axis represents the fluorescence intensity. This analysis demonstrates the positive colocalization of SHMT2 with HuD‐positive neurons in PFF‐injected and A53T mouse models. (J) Quantification of SHMT2‐positive staining area in the colon region of HuD‐positive stained sections across four groups. The bar graph shows SHMT2 upregulation in PFF‐injected and A53T mouse models. Values on the graphs are expressed as mean ± SEM; one‐way analysis of variance (ANOVA) was performed for (D, E, J), followed by Tukey's multiple comparisons test (***p < 0.001, ****p < 0.0001). IHC, immunohistochemistry; LOP, loperamide‐induced models; PFF, PFF‐induced models; SHAM, sham controls; SHMT2, serine hydroxymethyltransferase 2; SNc, substantia nigra pars compacta.

Representative images obtained from brain sections are presented in Figure 7A. Quantitative analysis of ROIs from brain sections revealed that the PFF group and A53T transgenic mice exhibited significantly elevated SHMT2 expression across all five samples, with IHC scores averaging 47.40 ± 4.63 and 42.12 ± 4.34, in comparison to the other two groups (Figure 7D). Similarly, in colon sections, the relative mean SHMT2 expression in PFF, LOP groups and A53T transgenic mice were observed to be significantly distinct from that noted in the SHAM group (Figure 7E).

Propagation of α‐syn pathology within the ENS, consistent with intracranial changes, constitutes a pivotal mechanism in clinical PD pathogenesis. To evaluate the pathological significance of SHMT2 upregulation in PFF‐induced neurodegeneration, SHMT2 distribution was mapped within the ENS of: (i) PFF‐injected mice at 6 months post‐injection and (ii) A53T transgenic mice at 3 months of age. Notably, SHMT2 immunofluorescence colocalized with the neuronal marker HuD in both PFF‐injected and A53T transgenic mice (Figure 7C,F–I). Quantification of SHMT2‐positive staining area further confirmed significant SHMT2 upregulation in colonic ENS lysates (Figure 7J). In contrast, in the loperamide‐induced constipation model, SHMT2 upregulation was restricted to the colonic glands and muscularis propria, with no significant increase observed within the ENS itself.

## 4 Discussion

Increasing evidence highlights the significance of linking constipation and PD. Constipation has been shown to be a significant predictor of PD, with studies suggesting that constipation may precede PD diagnosis by over a decade. Moreover, constipation has been shown to increase the risk of developing PD compared to healthy controls. Despite these findings, the pathological mechanisms underlying PD‐related constipation remain unclear. The development of constipation in PD patients likely involves a complex interplay of factors, including generalized hypokinesia, gut hypomotility, defecatory dysfunction, and the effects of anticholinergic and dopamine agonist medications [25]. While these factors contribute to PD progression, the mechanisms by which chronic constipation predisposes individuals to future PD remain poorly understood. Emerging evidence suggests that gut dysbiosis and enteric nervous system alterations may play critical roles in this relationship [26]. To enhance our understanding of the pathogenesis of the cooccurrence of constipation and PD, we utilized bioinformatics to analyze transcriptome data. Specifically, RNA sequencing expression profiles were employed to elucidate the transcriptional characteristics of PD and constipation, identifying shared DEGs. Subsequently, we conducted further validation and verification of the potential biomarkers using in vitro and in vivo models, including the PFF mouse model. These shared genes may serve as potential biomarkers for PD and constipation, as well as provide insights into the relationship between gene expression levels and PD progression.

Our study provides a deeper understanding of the different disease durations and molecular mechanisms underlying PD by using WGCNA analysis, which is vital in understanding deeply insights into the molecular mechanisms of PD progression. In order to intervene in the disease as early as possible and find potential therapeutic targets, we selected DEGs in PD putamen and caudate that were positively associated with the early stage of PD and upregulated in constipation for further screening. As a result, the yellow and pink module in the PD putamen exhibited a strong positive correlation with early‐stage disease, suggesting that the genes within this module may play a crucial role in the initial stages of PD. The enrichment analysis of these DEGs in the selected modules revealed an overrepresentation of genes involved in protein processing, as well as lipid metabolic processes and immune response, suggesting a complex interplay between these pathways in the pathophysiology of PD. Interestingly, previous studies have demonstrated that aberrant lipid levels and alterations in lipid metabolism can accelerate the accumulation of α‐syn, lead to mitochondrial and endoplasmic reticulum dysfunction, and trigger the activation of inflammatory and neuroinflammatory responses [27].

The transcriptional characteristics of PD and constipation identified in our study inform our understanding of the molecular mechanisms underlying PD, which we explore in the following sections. Notably, significant alterations in lipid metabolic pathways, including the carnitine shuttle and sphingolipid metabolism, have been observed in the sebum of PD patients, including both drug‐naïve and advanced patients [28], suggesting these changes may occur early in disease progression. This aligns closely with our findings that DEGs related to these pathways are positively correlated with early PD onset, reinforcing the importance of lipid metabolism in early pathogenesis. Notably, a significant upregulation of DEGs involved in protein folding and repair, as well as aberrant lipid metabolism, was observed in the constipation datasets, suggesting a potential link between the pathophysiology of constipation and the molecular mechanisms underlying PD, and highlighting the need for further investigation into the relationship between these two conditions. A recent study demonstrated that glial α‐syn expression in a Drosophila model leads to significant changes in lipid metabolic pathways and protein folding mechanisms [29], similar to those observed in our study. Dysregulation of lipid metabolism as well as the accumulation of α‐syn may trigger a cascade of cellular events, including mitochondrial and endoplasmic reticulum dysfunction, which can have far‐reaching consequences for cellular homeostasis [26, 30]. This, in turn, suggests that the interplay between these pathways may be a common underlying mechanism in the pathophysiology of PD, affecting both central and peripheral manifestations of the disease.

In the context of PD caudate, the turquoise and yellow modules were the only ones to exhibit positive correlations with early‐stage disease, albeit with relatively weak correlations. The turquoise module, characterized by a pronounced positive correlation with early stage in PD caudate, was further selected and found to be predominantly associated with potential aberrations in DNA repair mechanisms and protein homeostasis, which may have far‐reaching implications for cellular integrity. This finding is supported by previous studies demonstrating that oxidative stress and DNA damage are critical factors in the pathogenesis of early‐stage PD, particularly in regions such as the caudate nucleus [11]. Specifically, the accumulation of DNA strand breaks and alterations in DNA conformation observed in the caudate nucleus of PD brains suggest that impaired DNA repair mechanisms could contribute to neuronal vulnerability in this region [31]. Additionally, the caudate nucleus is known to be susceptible to oxidative stress [32], which can disrupt protein homeostasis and lead to the accumulation of misfolded proteins, further compromising cellular integrity. Therefore, the observed correlation between the turquoise module and early‐stage PD in the caudate nucleus may reflect an underlying disruption in these critical cellular processes, highlighting the importance of investigating DNA repair and protein homeostasis as potential therapeutic targets in early‐stage of PD. Notably, KEGG analysis revealed adjusted *p*‐values > 0.05, potentially due to limited statistical power from the modest sample size. This finding may be attributed to the small sample size or complex PD pathology, introducing variability in gene expression profiles. Future investigations with increased sample sizes and improved methodologies are necessary to validate results and elucidate PD mechanisms.

To further explore the relationship between constipation and PD, we utilized the PFF mouse model, which has been shown to replicate key aspects of PD pathology. The intracranial injection of PFFs in mice effectively induces both motor dysfunction and gastrointestinal symptoms, such as constipation, thus providing a comprehensive model that closely mimics the multisystem pathology of Parkinson's disease [24, 33]. This model offers a valuable tool for studying the pathogenesis of PD and evaluating potential therapeutic strategies targeting both motor and non‐motor symptoms. Our findings revealed that the PFF model exhibits characteristic motor dysfunction and gastrointestinal abnormalities, including altered fecal properties and prolonged colonic transit time. This is consistent with previous studies and can be attributed to impaired colonic motility [8], validating the model's utility in replicating PD pathology. Complementarily, A53T transgenic mice (bearing a familial PD‐associated mutation) were leveraged to authenticate SHMT2's PD relevance. This genetic model complements PFF systems by interrogating hereditary PD mechanisms.

The IHC analysis of midbrain sections demonstrated markedly upregulated SHMT2 expression in PFF‐injected and A53T transgenic mice relative to controls. Our results are in accordance with existing literature, which highlights the involvement of SHMT2 in various neurodegenerative and metabolic disorders, underscoring its potential role as a key regulator. SHMT2, a mitochondrial enzyme involved in one‐carbon metabolism, plays a crucial role in the transfer of one‐carbon units, which are essential for nucleotide synthesis, DNA and protein methylation, and mitochondrial metabolism. According to previous studies [34], mutations in PINK1 and PARKIN, two critical regulators of mitochondrial quality control, have been shown to induce mitochondrial dysfunction and concurrently upregulate SHMT2 expression in Drosophila models of PD, thereby highlighting the complex interplay between mitochondrial function and one‐carbon metabolism. In vitro, SHMT2 knockdown via siRNA significantly attenuated PFF‐induced mitochondrial damage and oxidative stress. Parallel mechanisms exist in Alzheimer's disease, where mitochondrial one‐carbon dysregulation amplifies oxidative stress and genomic instability [35]. Physiologically, SHMT2 elevation may sustain neuronal homeostasis by augmenting nucleotide pools and mitochondrial biogenesis—potentially counteracting neurodegeneration. Conversely, pathological SHMT2 overexpression disrupts serine‐glycine stoichiometry, exacerbating oxidative stress and mitophagy failure [36]. We thus propose that early‐stage PD features SHMT2 upregulation as a compensatory response to mitochondrial damage and apoptotic stress. Whether this represents adaptive neuroprotection or contributes to pathogenic cascades requires longitudinal assessment of SHMT2 spatiotemporal dynamics.

Our findings also suggest that SHMT2 may play a role in the gastrointestinal manifestations, as evidenced by the significant difference in SHMT2 expression among the PFF, LOP groups, transgenic mice, and the SHAM group in the colon. This pattern indicates a potential role for SHMT2 and one‐carbon metabolism in gastrointestinal dysfunction. Previous studies have shown that hyperhomocysteinemia, characterized by elevated one‐carbon metabolite activity, can impair intestinal motility, trigger inflammatory responses, and cause constipation [37], with broad implications for gut health. Additionally, short‐term methionine supplementation has been shown to influence one‐carbon metabolism and DNA methylation in the gut, leading to alterations in the gut microbiome, barrier function, gene expression, and histomorphology [38]. This suggests that changes in one‐carbon metabolism can have broad effects on gut function. The disruption of the gut‐brain axis, potentially caused by this phenomenon, highlights the intricate relationships between one‐carbon metabolism, gastrointestinal health, and neurodegenerative disease pathology. Our results also indicate that shared DEGs enriched in PD and constipation are significantly related to one‐carbon metabolism, further supporting the link between one‐carbon metabolism and gastrointestinal health. Interestingly, elevated SHMT2 in PFF models within gut neurons might reflect disease‐driven interactions between the brain and gut, where pathological protein aggregates affect both the brain and ENS. Moreover, changes in one‐carbon metabolism, particularly the methionine cycle, can regulate gut physiological functions and behavior through neuron‐gut signaling [39]. However, in the loperamide‐induced constipation model, the lack of significant SHMT2 elevation suggests that gut neurons may not be activated in the same way as in PD models. This implies that drug‐induced constipation models may not fully capture the non‐motor symptoms of PD, possibly due to the absence of the complex interplay between the gut and brain that is characteristic of PD pathology. Overall, SHMT2 and one‐carbon metabolism may represent potential therapeutic targets for the treatment of constipation, particularly in PD patients where both gastrointestinal and neurological manifestations are present. Future studies should employ larger sample sizes and advanced techniques such as genomics, proteomics, and metabolomics to further explore the molecular mechanisms underlying one‐carbon metabolism and its role in PD‐related gastrointestinal dysfunction. This comprehensive approach will be crucial for identifying effective therapeutic strategies.

Despite the valuable insights provided by our study, several limitations should be acknowledged. First, the transcriptome analysis, particularly of dataset GSE101968, involved a small sample size. This limitation may affect the statistical power and the generalizability of our findings. Future studies should include larger cohorts to validate our results and enhance the robustness of the identified biomarkers. Secondly, although our in vivo model successfully recapitulated key aspects of PD pathology, it is likely that this model does not fully capture the intricate complexity and heterogeneity of human disease. For instance, creating models with ENS‐specific α‐syn overexpression or SHMT2 knockdown could offer more detailed insights. Thus, further studies in human subjects are necessary. These studies will help to confirm the clinical relevance and translational potential of our findings. Furthermore, longitudinal studies are essential to elucidate the temporal dynamics of SHMT2 regulation and its relationship to disease progression, as this will provide critical insights into the underlying mechanisms and facilitate the development of more effective therapeutic strategies.

## 5 Conclusion

Our study provides valuable insights into the molecular mechanisms linking PD and constipation, identifying shared biological processes and SHMT2 as a potential biomarker. The findings from our comprehensive transcriptome analysis and in vivo validations highlight the potential of SHMT2 as a therapeutic target for both motor and non‐motor symptoms of PD. Future research should focus on further elucidating the role of SHMT2 and the underlying molecular mechanisms in PD‐related constipation pathogenesis, as well as exploring their potential as diagnostic and therapeutic tools.

## Author Contributions

J.S. and X.J. were involved in the conception and design of the study; K.H., C.W., R.G., and Z.R. were involved in the acquisition of the data; J.S., X.J., X.L., and Z.L. curated the data; J.S., X.J., X.L., D.L., and E.T. analyzed and interpreted the data; Y.Z. performed data analysis and statistical processing; J.S. and K.H. drafted the article; X.L., D.L., and E.T. critically revised for intellectual content; and all authors revised and approved the final manuscript.

## Funding

This research was funded by Shenzhen Science and Technology Program (JCYJ20220818102206014), and the National Natural Science Foundation of China (82271454).

## Ethics Statement

This study was approved by the Sun Yat‐Sen University (Approval number: SYSU‐IACUC‐2024‐002260), and all methods were performed in accordance with the relevant guidelines and regulations.

## Consent

The authors have nothing to report.

## Conflicts of Interest

The authors declare no conflicts of interest.

## Supporting information


**Table S1:** Table of primer sequence for qRT‐PCR used in this study.
**Table S2:** Primers for SHMT2 Silencer Select siRNA used in this study.
**Table S3:** Results of KEGG pathway enrichment in turquoise module.

## Data Availability

The data that support the findings of this study are available on request from the corresponding author. The data are not publicly available due to privacy or ethical restrictions.
