# Supplementary material for: Novel Insights Into the Association Between Parkinson's Disease and Constipation: Role of SHMT2 as a Promising Biomarker
Source: CNS Neurosci Ther. 2026 May 3;32(5):e70912. doi: 10.1002/cns.70912 (PMC13136694; doi:10.1002/cns.70912)
Supplement: Supplementary file 1 — Table S1: Table of primer sequence for qRT‐PCR used in this study. Table S2: Primers for SHMT2 Silencer Select siRNA used in this study. Table S3: Results of KEGG pathway enrichment in turquoise module. [file CNS-32-e70912-s001.docx]

Description of Additional Supplementary Data Files

Table S1 Table of primer sequence for qRT-PCR used in this study.

Table S2 Primers for SHMT2 Silencer Select siRNA used in this study.

Table S3 Results of KEGG pathway enrichment in turquoise module

Table S1 Primer sequence for qRT-PCR.

| Target genes | Forward sequence (5’ - 3’) | Reverse sequence(5’ - 3’) | Species |
| --- | --- | --- | --- |
| GAPDH | GAGAAGGCTGGGGCTCATTTG | CAGGAGGCATTGCTGATGATC | Human |
| SHMT2 | CGGACATCGTCACCACCACT | GGCAAAGTTGATTCGGTCCT | Human |
| MTHFD2 | AGGACGAATGTGTTTGGATCAG | GGAATGCCAGTTCGCTTGATTA | Human |
| HSPE1 | TGGCAGGACAAGCGTTTAGA | CCGATCCAACAGCGACTACT | Human |
| HSPD1 | TGGAGTGGCTGTGCTGAAGG | CACAACCCCCTCCCAAAACA | Human |
| RCC1 | GGAGATGGTCCCTGGGAAAG | CCAACAGTCCAATCACACCG | Human |

Table S2 Primer sequence for SHMT2 Silencer Select siRNA.

| Names | sequence (5’ - 3’) | Species |
| --- | --- | --- |
| Primers for SHMT2 forward | GCGCCUAUGCUCGCCUCAUTT | Human |
| Primers for SHMT2 reverse | AUGAGGCGAGCAUAGGCGCTT | Human |

Table S3 KEGG pathway enrichment in turquoise module

| **ID** | **category** | **Description** | **GeneRatio** | **BgRatio** | **pvalue** | **p.adjust** |
| --- | --- | --- | --- | --- | --- | --- |
| hsa04110 | Cellular Processes | Cell cycle | 30/911 | 158/8541 | 1.16E-03 | 1.72E-01 |
| hsa01210 | Metabolism | 2-Oxocarboxylic acid metabolism | 10/911 | 33/8541 | 1.70E-03 | 1.72E-01 |
| hsa00310 | Metabolism | Lysine degradation | 15/911 | 63/8541 | 2.10E-03 | 1.72E-01 |
| hsa03440 | Genetic Information Processing | Homologous recombination | 11/911 | 41/8541 | 2.98E-03 | 1.72E-01 |
| hsa00020 | Metabolism | Citrate cycle (TCA cycle) | 9/911 | 30/8541 | 3.10E-03 | 1.72E-01 |
| hsa00280 | Metabolism | Valine, leucine and isoleucine degradation | 12/911 | 48/8541 | 3.71E-03 | 1.72E-01 |
| hsa04120 | Genetic Information Processing | Ubiquitin mediated proteolysis | 26/911 | 142/8541 | 4.02E-03 | 1.72E-01 |
| hsa03460 | Genetic Information Processing | Fanconi anemia pathway | 13/911 | 55/8541 | 4.35E-03 | 1.72E-01 |
| hsa00603 | Metabolism | Glycosphingolipid biosynthesis - globo and isoglobo series | 6/911 | 16/8541 | 4.52E-03 | 1.72E-01 |
| hsa00640 | Metabolism | Propanoate metabolism | 9/911 | 32/8541 | 4.99E-03 | 1.72E-01 |
| hsa04141 | Genetic Information Processing | Protein processing in endoplasmic reticulum | 29/911 | 170/8541 | 7.00E-03 | 2.19E-01 |
| hsa05202 | Human Diseases | Transcriptional misregulation in cancer | 32/911 | 198/8541 | 1.06E-02 | 3.05E-01 |
| hsa03420 | Genetic Information Processing | Nucleotide excision repair | 13/911 | 63/8541 | 1.41E-02 | 3.33E-01 |
| hsa00785 | Metabolism | Lipoic acid metabolism | 6/911 | 20/8541 | 1.51E-02 | 3.33E-01 |
| hsa05131 | Human Diseases | Shigellosis | 38/911 | 250/8541 | 1.52E-02 | 3.33E-01 |
| hsa03022 | Genetic Information Processing | Basal transcription factors | 10/911 | 44/8541 | 1.55E-02 | 3.33E-01 |
| hsa05017 | Human Diseases | Spinocerebellar ataxia | 24/911 | 144/8541 | 1.75E-02 | 3.54E-01 |
| hsa04140 | Cellular Processes | Autophagy - animal | 27/911 | 169/8541 | 2.06E-02 | 3.94E-01 |
| hsa04115 | Cellular Processes | p53 signaling pathway | 14/911 | 75/8541 | 2.57E-02 | 4.50E-01 |
| hsa04218 | Cellular Processes | Cellular senescence | 25/911 | 157/8541 | 2.62E-02 | 4.50E-01 |
| hsa04920 | Organismal Systems | Adipocytokine signaling pathway | 13/911 | 70/8541 | 3.21E-02 | 5.25E-01 |
| hsa04975 | Organismal Systems | Fat digestion and absorption | 9/911 | 43/8541 | 3.48E-02 | 5.42E-01 |
| hsa01240 | Metabolism | Biosynthesis of cofactors | 24/911 | 154/8541 | 3.62E-02 | 5.42E-01 |
| hsa03082 | Genetic Information Processing | ATP-dependent chromatin remodeling | 19/911 | 117/8541 | 4.05E-02 | 5.80E-01 |
| hsa00270 | Metabolism | Cysteine and methionine metabolism | 10/911 | 52/8541 | 4.55E-02 | 6.26E-01 |
| hsa04136 | Cellular Processes | Autophagy - other | 7/911 | 32/8541 | 4.80E-02 | 6.35E-01 |
| hsa04137 | Cellular Processes | Mitophagy - animal | 17/911 | 105/8541 | 5.19E-02 | 6.38E-01 |
| hsa04210 | Cellular Processes | Apoptosis | 21/911 | 136/8541 | 5.20E-02 | 6.38E-01 |
| hsa04146 | Cellular Processes | Peroxisome | 14/911 | 83/8541 | 5.50E-02 | 6.52E-01 |
| hsa05014 | Human Diseases | Amyotrophic lateral sclerosis | 49/911 | 371/8541 | 6.54E-02 | 7.50E-01 |
| hsa00601 | Metabolism | Glycosphingolipid biosynthesis - lacto and neolacto series | 6/911 | 28/8541 | 7.08E-02 | 7.61E-01 |
| hsa00790 | Metabolism | Folate biosynthesis | 6/911 | 28/8541 | 7.08E-02 | 7.61E-01 |
| hsa04217 | Cellular Processes | Necroptosis | 23/911 | 159/8541 | 7.95E-02 | 8.05E-01 |
| hsa00330 | Metabolism | Arginine and proline metabolism | 9/911 | 50/8541 | 7.96E-02 | 8.05E-01 |
| hsa03030 | Genetic Information Processing | DNA replication | 7/911 | 36/8541 | 8.24E-02 | 8.10E-01 |
| hsa00910 | Metabolism | Nitrogen metabolism | 4/911 | 17/8541 | 9.94E-02 | 8.94E-01 |
| hsa04623 | Organismal Systems | Cytosolic DNA-sensing pathway | 13/911 | 83/8541 | 1.00E-01 | 8.94E-01 |
| hsa00630 | Metabolism | Glyoxylate and dicarboxylate metabolism | 6/911 | 31/8541 | 1.06E-01 | 8.94E-01 |
| hsa01200 | Metabolism | Carbon metabolism | 17/911 | 116/8541 | 1.09E-01 | 8.94E-01 |
| hsa05168 | Human Diseases | Herpes simplex virus 1 infection | 25/911 | 182/8541 | 1.11E-01 | 8.94E-01 |
| hsa00670 | Metabolism | One carbon pool by folate | 7/911 | 39/8541 | 1.16E-01 | 8.94E-01 |
| hsa04150 | Environmental Information Processing | mTOR signaling pathway | 22/911 | 158/8541 | 1.16E-01 | 8.94E-01 |
| hsa00061 | Metabolism | Fatty acid biosynthesis | 4/911 | 18/8541 | 1.18E-01 | 8.94E-01 |
| hsa03040 | Genetic Information Processing | Spliceosome | 29/911 | 217/8541 | 1.18E-01 | 8.94E-01 |
| hsa05132 | Human Diseases | Salmonella infection | 33/911 | 251/8541 | 1.19E-01 | 8.94E-01 |
| hsa05016 | Human Diseases | Huntington disease | 40/911 | 311/8541 | 1.20E-01 | 8.94E-01 |
| hsa00620 | Metabolism | Pyruvate metabolism | 8/911 | 47/8541 | 1.22E-01 | 8.94E-01 |
